# Study on collagen parameters in vulvar cancer and preneoplastic lesions by Second Harmonic Generation microscopy

**DOI:** 10.1038/s41598-020-62346-8

**Published:** 2020-03-27

**Authors:** Maria das Graças de Fátima Cavalcanti Castor, Leuridan Cavalcante Torres, Roberto José Vieira de Mello, Rodrigo de Andrade Natal, José Vassallo

**Affiliations:** 1 Division of Pelvis, Hospital de Cancer de Pernambuco, Recife, PE, Brazil; 2 Translational Research Laboratory C. A. Hart, Instituto de Medicina Integral Prof. Fernando Figueira (IMIP), Recife, PE Brazil; 3 Division of Anatomic Pathology, Hospital de Cancer de Pernambuco, Recife, PE, Brazil; 4 Laboratory of Investigative Pathology, CIPED, Faculdade de Ciências Médicas, UNICAMP, Campinas, SP Brazil; 5 Department of Anatomic Pathology, AC Camargo Cancer Center, São Paulo, Brazil

**Keywords:** Oncology, Cancer, Gynaecological cancer

## Abstract

The extracellular matrix plays an important role in cellular balance, and collagen fibers are its most important component. Over the last few years, second harmonic generation (SHG) microscopy has been used for the analysis of collagen fibers in several types of gynaecological cancers, such as breast and ovarian cancer. The value of collagen parameters obtained with this technique to gain insights on the physiopathology and on the prognostic evaluation of cancer has been advocated. Herein, we have characterized the collagen fibers in squamous cell carcinoma (VSCC) and preneoplastic lesions using the SHG microscopy. Collagen parameters, quantity, organization, and uniformity, of VSCC, adjacent skin of VSCC or preneoplastic lesions were compared with values obtained in normal tissue of healthy control. There was an evident decrease in the values of collagen fiber parameters in the VSCC. Increased quantity and uniformity of tumor associated collagen fibers were associated with the presence of lymph node metastases, which suggest a prognostic value of such parameters in the evaluation of vulvar cancer.

## Introduction

Vulvar cancer is the fourth leading gynaecological cancer worldwide^1^, following uterine corpus, ovarian, and cervical cancer. It represents only about 5% of all tumors of the lower genital tract^1,2^ and is thus considered a rare cancer. Among the different histological types of vulvar cancer, squamous cell carcinoma (VSCC) is the most common type (95%), followed by melanoma (<5%), sarcoma (1–2%), and basal-cell carcinoma (2%). VSCC has two mechanisms of development, which arise via preneoplastic lesions called vulvar intraepithelial neoplasia (VIN): (1) infection with the human papillomavirus (HPV), which affects younger women (<50 years), and (2) HPV-independent development (approximately 80%), which appears after menopause (50–80 years) and originates from lichen sclerosus^3–6^.

VIN is graded in a similar way as cervical intraepithelial neoplasia: VIN 1, VIN 2, or VIN 3. In VIN 1, atypical cells are limited to the lower third of the vulvar squamous epithelium; in VIN 2, atypical cells compromise the deeper two thirds of the epithelium; and in VIN 3, atypical cells affect the entire squamous epithelium. In all three grades, the basal membrane remains intact, i.e., the lesions are not invasive. VIN 1 is not considered a precursor of vulvar cancer, as it has low malignant potential and low probability of evolving into VIN 2 and VIN 3. These last two grades instead are considered high-grade lesions with real risk to transform into malignancy^7,8^.

Over the last few years, studies on cancer development have focused on the role of remodeling of the extracellular matrix (ECM), more specifically on collagen fibers (COL), to tumor progression. COL are major components of the ECM and are important for the maintenance of tissue and organ structure and integrity^9,10^. However, its role surpasses the structural aspect, and it is considered to influence tumor invasiveness and metastasizing^9,10^.

The study of COL in histology laboratories goes from the traditional stains, such as Masson’s trichrome, Movat pentachrome, and picrosirius red staining, to the more specific detection methods, as immunohistochemistry, the wide-field-of-view polarization, and the second harmonic generation (SHG) imaging. The SHG has been increasingly being used to study COL, due to its high sensitivity and specificity. It is a quantitative nonlinear optical microscopy technique that captures images from biological tissues having polarized anisotropic structures, such as COL^11,12^.

The association of increased COL expression in vulvar carcinoma and higher tumor aggressiveness has been recently reported, but the evaluation of COL was made using the picrosirius staining^13^. In the present study, SHG images were used to determine the quantity, uniformity, and organization of COL in vulvar cancer and its preneoplastic lesions. It is intended to determine the clinical value of COL evaluation by this method, not previously reported in vulvar neoplasia.

## Results

### Collagen fiber parameters in VSCC

**Figure 1:**
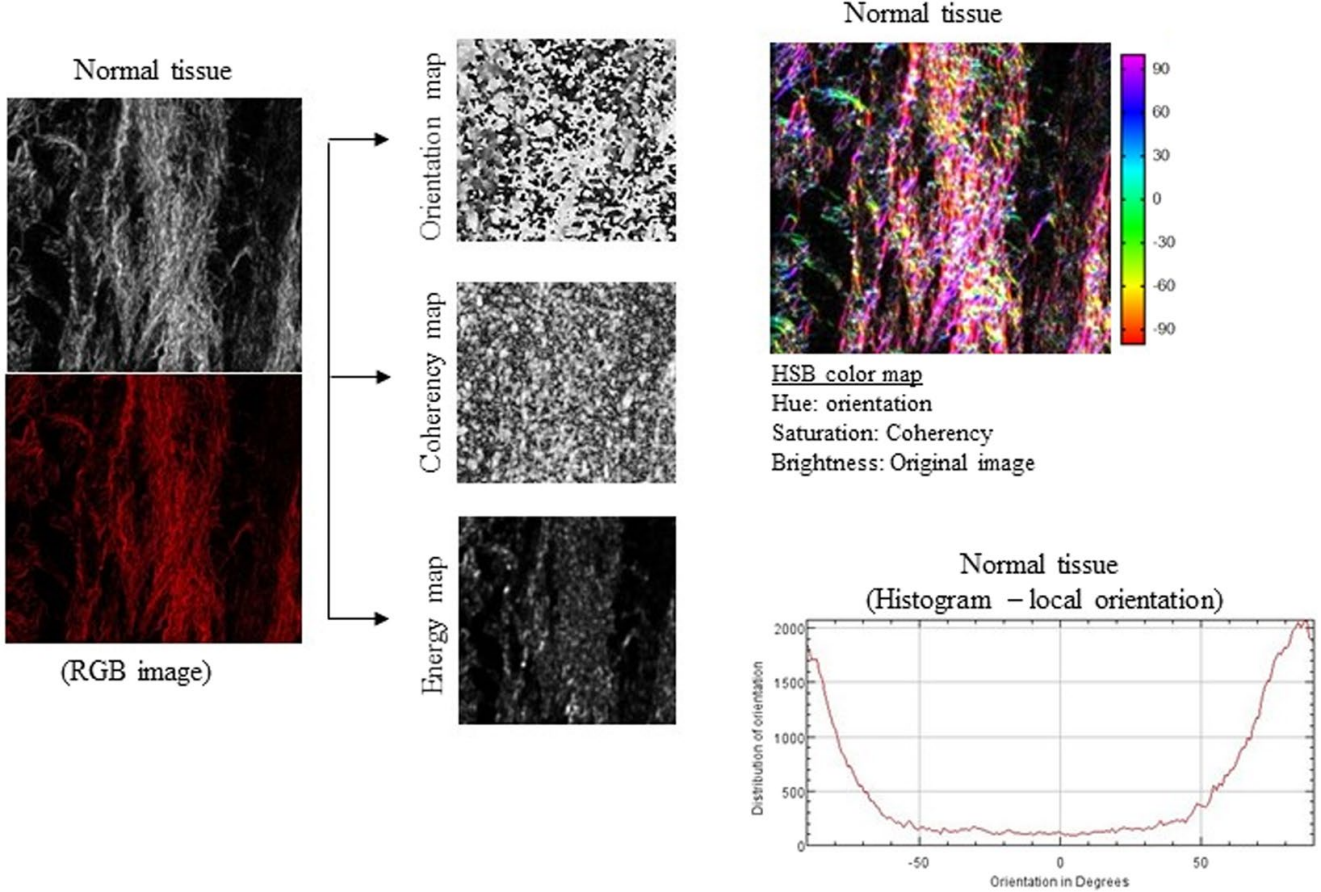
Results of the OrientationJ, the imageJ plug-in developed to get the histogram of local angles, the orientation, energy (quantity) and coherency (uniformity) maps of an image and the HSB color-coded map of normal tissue of healthy control.

**Figure 2:**
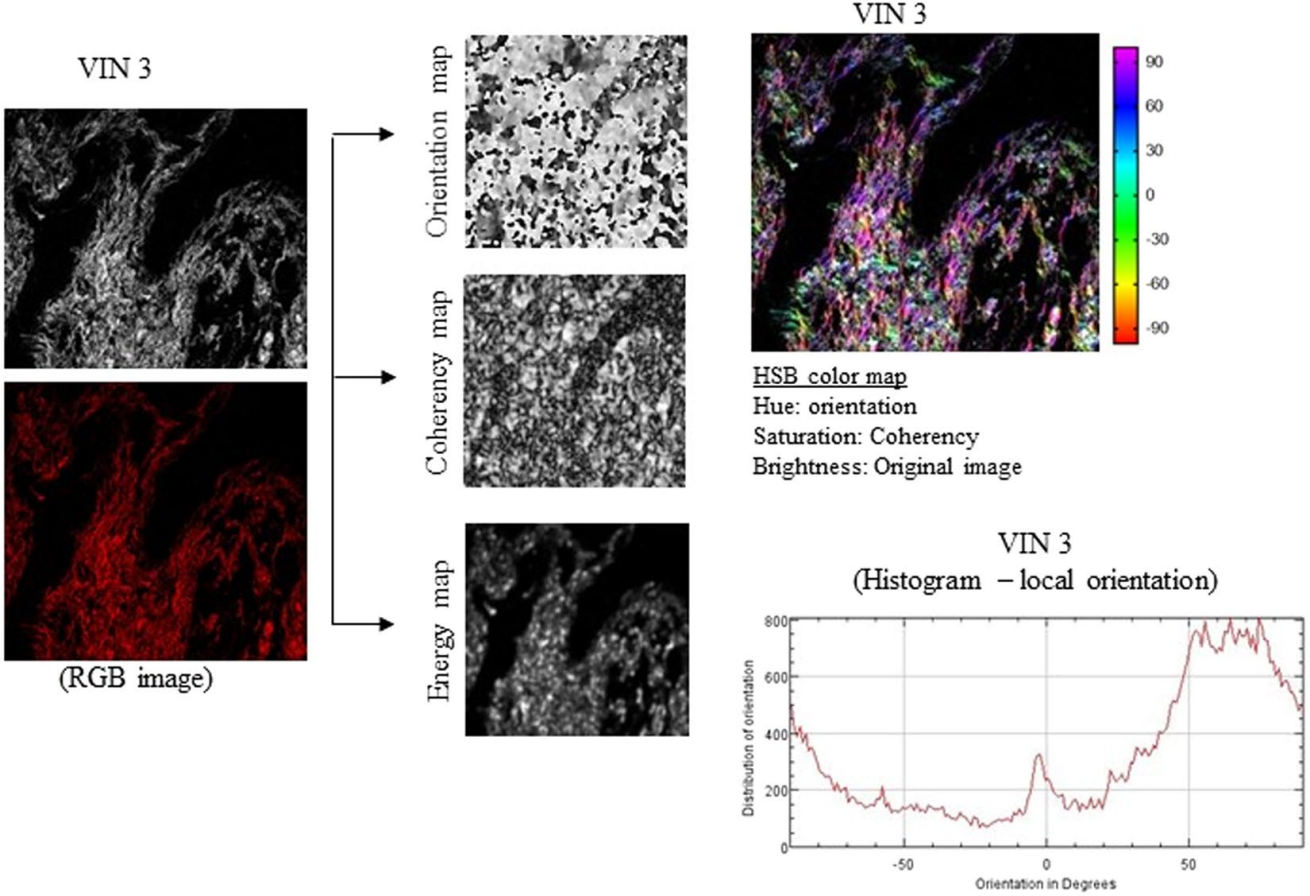
Results of the OrientationJ, the imageJ plug-in developed to get the histogram of local angles, the orientation, energy (quantity) and coherency (uniformity) maps of an image and the HSB color-coded map of VIN 3.

**Figure 3:**
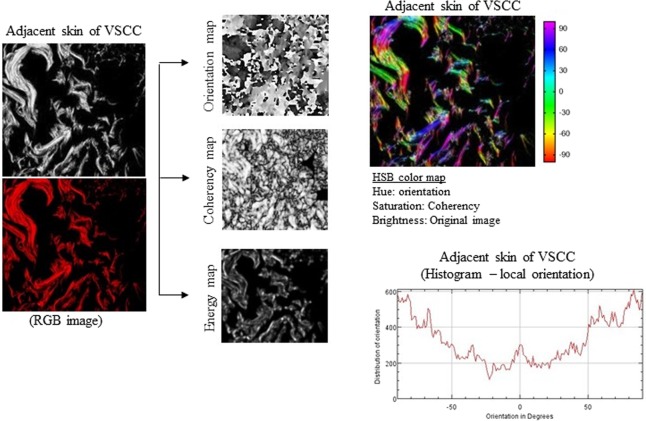
Results of the OrientationJ, the imageJ plug-in developed to get the histogram of local angles, the orientation, energy (quantity) and coherency (uniformity) maps of an image and the HSB color-coded map of adjacent skin of vulvar squamous cell carcinoma (VSCC).

**Figure 4:**
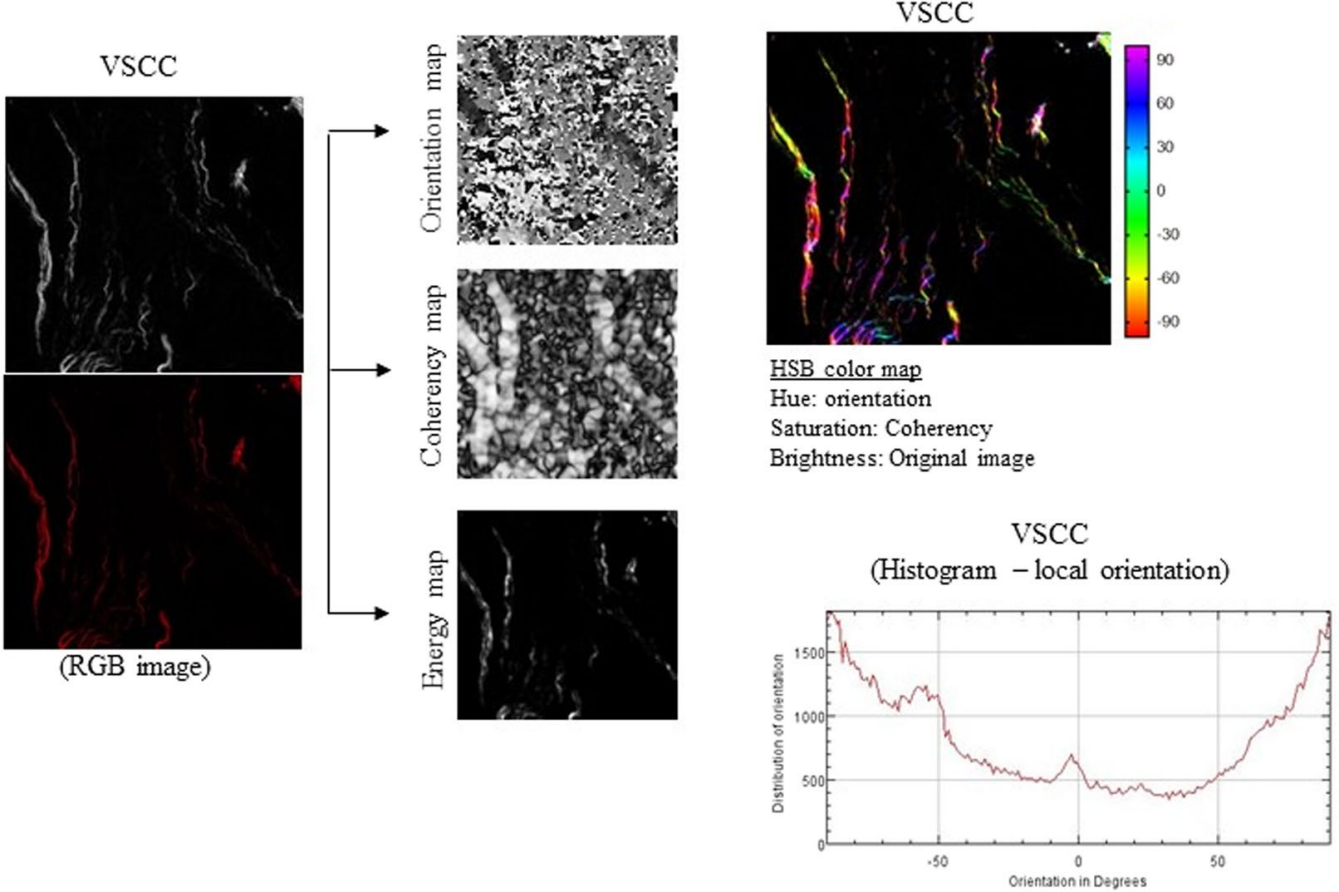
Results of the OrientationJ, the imageJ plug-in developed to get the histogram of local angles, the orientation, energy (quantity) and coherency (uniformity) maps of an image and the HSB color-coded map of vulvar squamous cell carcinoma (VSCC).

**Figure 5:**
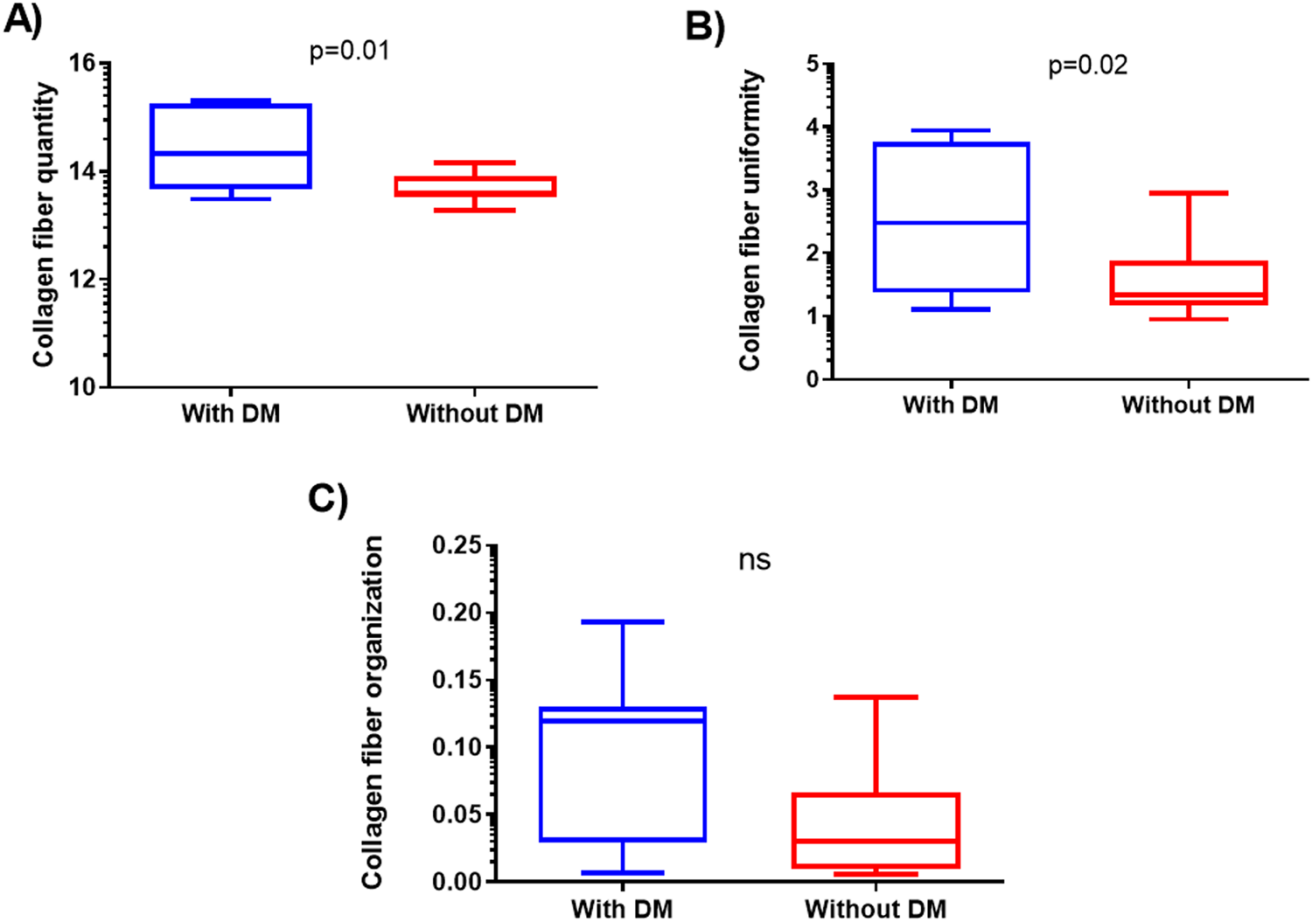
Comparative analysis of collagen parameters in vulvar squamous cell carcinoma (VSCC) in patients with distant metastasis (DM) and without DM: (**A**) Collagen Fiber quantity, (**B**) Collagen Fiber uniformity and (**C**) Collagen Fiber organization. Ns: non-significant.

We made the OrientationJ plug-in and an ImageJ for angle analysis the orientation, energy, and coherency maps, hue-saturation-brightness (HSB) color-coded map and orientation histogram in normal tissue of healthy control (individual without VSCC or VIN; Figure 1), VIN 3 (Figure 2) and adjacent skin of VSCC (Figure 3) and VSCC (Figure 4). Images show the number of collagen fibers arranged at different angles and tissues. It is observed that normal tissue of healthy control presents the better organization of collagen fibers, demonstrated by the peak between angles 60° and 90° (Figure 1). In the analysis of VIN 3 and adjacent skin of VSCC, we observed the highest amount of collagen fibers compared to VSCC (Figures 2 and 3, respectively). It is observed peaks in the number of collagen fibers at various angles in VSCC, showing that the region presents an absence of collagen fibers organization (Figure 4). Collagen quantity and uniformity parameters were significantly higher in group of VSCC patients with distant metastatic compared to without metastasis (p = 0.01 and p = 0.02, respectively; Figure 5).

The analysis of the collagen fibers revealed decreased quantity, uniformity, and organization in intratumoral stroma compared to adjacent skin of VSCC (p < 0.05; Table 1). According to the tumoral topography (labia minora, labia majora, and both), the quantity, uniformity, and organization of collagen parameters of labia minora were decrease compared to labia majora and in both labia (p = 0.008, p = 0.01, and p = 0.005, respectively) (Table 1). The quantity, uniformity, and organization of collagen fibers were reduced in the intratumoral stroma when compared to adjacent skin in labia minora and labia majora in the VSCC (p < 0.05; Table 2).

**Table 1:** Collagen fibers parameters of intratumoral area and adjacent skin of vulvar squamous cell carcinoma (VSCC), and according to the tumoral topography.

Parameters	VSCC		P-valor*	Topography			P-valor**
	Intratumoral stroma	adjacent skin		Labia minora	Labia majora	In both labia	
	Median (IQR)	Median (IQR)		Median (IQR)	Median (IQR)	Median (IQR)	
Quantity	13.90 (13.55–15.10)	16.10 (15.73–16–48)	<0.0001	13.53 (13.4–13.8)	14.43 (13.7–15.3)	14.13 (13.6–15.9)	0.008
Uniformity	1.90 (1.25–3.60)	4.61 (4.01–5.28)	<0.0001	1.22 (1.09–1.70)	2.55 (1.42–3.68)	2.32 (1.30–4.21)	0.011
Organization	0.064 (0.014–0.17)	0.21 (0.11–0.24)	0.03	0.02 (0.003–0.06)	0.10 (0.02–0.19)	0.05 (0.02–0.10)	0.005

IQR: interquartile; *Mann Whitney test; **Kruskal-Wallis test. p < 0.05.

**Table 2:** Collagen fibers parameters of intratumoral stroma and adjacent skin of vulvar squamous cell carcinoma (VSCC) in the labia minora and labia majora.

Parameters	VSCC - Labia minora		P-valor*	VSCC - Labia majora		P-valor*
	Intratumoral stroma	adjacent skin		Intratumoral stroma	adjacent skin	
	Median (IQR)	Median (IQR)		Median (IQR)	Median (IQR)	
Quantity	13.54 (13.4–14.02)	15.73 (15.6–17.1)	0.001	14.43 (13.6–15.3)	16.7 (16.1–16.4)	<0.0001
Uniformity	1.25 (1.09–1.90)	4.06 (3.94–5.89)	0.0003	2.55 (1.42–3.68)	4.68 (4.55–5.22)	0.0005
Organization	0.033 (0.006–0.054)	0.17 (0.14–0.29)	0.001	0.10 (0,02–0.19)	0.21 (0.20–0.24)	0.004

IQR: interquartile; *Mann Whitney test. p < 0.05.

## Discussion

COL parameters are reduced in VSCC and VIN in comparison to dermis adjacent to neoplasia. However, no significant difference was observed comparing COL parameters between VSCC and VIN patients. Additionally, patients with metastatic VSCC presented higher COL parameters than those without metastases. These findings agree with our previous study on luminal breast cancer, in which intratumoral COL parameters were reduced in comparison to peritumoral areas^14^. Similarly, patients with parameters associated with more aggressive disease, presented higher intratumoral COL values^14^. Using another method to evaluate COL expression, it was recently found that higher values in VSCC were associated to superior aggressiveness^12^.

SHG microscopy has been used over the last few years to analyze COL in several types of cancer, due to its high sensitivity and specificity^11,12,15^. In our study, we used SHG to study COL in vulvar cancer, which, although considered a rare cancer of elderly women, has been increasing in incidence in younger women over the last few decades^16–18^. Studies on breast, head and neck, esophageal, and colorectal cancer using SHG reported that longer and denser COL in the tumor microenvironment was associated with tumor invasion^9,10^. Others have shown that the presence of tumor invasion was associated with higher COL quantity and uniformity, but lower organization^19,20^.

When comparing lesions according to the anatomical site, the labia minora showed a decreased quantity, uniformity, and organization of intratumor COL in relation to the labia majora. This difference might be due to the heterogeneous composition of both tissues, one more akin to the mucosa and the other, to the skin.

The incidence of vulvar cancer is highest in elderly, post-menopausal women, in whom decreased estrogen production may be associated with a negative effect on cutaneous turgor, loss of skin elasticity^21^ and reduced immune defences^22,23^. It could be hypothesized that the recurrent inflammation due to these predisposing factors might play a role in the development of vulvar cancer. Although the tissues adjacent to our patient’s tumors by histological examination were considered tumor-free margins, it was observed that on visualization of images obtained by SHG microscopy, these tissues showed small changes in COL parameters when compared to normal tissue in healthy women. Normal tissue was used for comparative analysis of the normal tissue image adjacent to the tumor by microscopy. We know that the free margin of the tumor is very important for locoregional control of the vulvar tumor, because when compromised, it is usually indicative for performing a re-excision^24^. This suggests that SHG microscopy may be an important meteorology for safely distinguishing areas that are tumor-free margins from those with very early precursor lesions.

The present study has some limitations. The number of cases is relatively small, due the uni-institutional design. However, it represents an infrequent cancer, and encouraging data were obtained on COL quantification in this setting. Another limitation is represented by the fact that the controls used were areas in the same samples, without microscopically detectable neoplasia. There is a potential that finer alterations at the molecular level could have interfered with the results, but a significant difference has been achieved between most parameters from tumor versus non-tumor areas. The use of SHG can be considered a drawback, because of the costs of the equipment and difficulty in managing it. However, it should be acknowledged that this cost has been decreasing over the last few years, what may allow SHG to become included in the diagnostic practice in the near future^11,12^.

Our findings on vulvar neoplasia add information on COL quantification by SHG microscopy in vulvar carcinoma and corroborate the general results with other tumors studied. They reinforce the notion that COL quantity and distribution play an important role in tumor progression. Further, it is shown herein that alterations in COL are precocious in vulvar carcinogenesis, as the same alterations were found already in VIN.

## Methods

### Experimental design and participants

An analytical and retrospective study was performed between 2000 and 2010, after approval by the Human Research Ethics Committee of HCP – Number of Certificate of Presentation for Ethical Appreciation is 50720115.9.0000.5205. The guidelines followed the resolution number 466/12 of the National Health Council of Brazil for involving human participants. The informed consent was obtained from all subjects. The study was executed at the Cancer Hospital of Pernambuco (HCP, Pernambuco), and the Multiuser Laboratory of the “Gleb Wataghin” Physics Institute (INFABiC - State University of Campinas - Unicamp, São Paulo). A survey was undertaken on patient records with the diagnoses of VSCC and VIN, between 2000 and 2010. The formalin fixed paraffin embedded tissue blocks from the patients with both diagnoses were retrieved from the archives of the Pathology Department of Hospital de Cancer de Pernambuco, Recife, Brazil.

The study included a total of 52 Brazilian patients older than 18 years, of which 40 patients had been diagnosed with VSCC and 12 with potential precursor lesions (VIN 2 and VIN 3). Adjacent tissues to the tumor with no neoplastic and/or preneoplastic morphological features were used as controls (17 patients). The metastases considered in the study were all to the lymph nodes, confirmed by histopathology of the surgical specimens (seven patients; Table 3). Clinicopathological data were obtained from the patients’ files and from the pathological reports, and included age, topography, and presence of metastases (Table 3). All H&E stained slides were reviewed by the pathologists and the areas of interest were selected for studies using the second harmonic generation microscopy (SHG).

**Table 3:** Clinical characteristics of patients with vulvar squamous cell carcinoma (VSCC) and vulvar intraepithelial neoplasia (VIN).

PARAMETERS	VSCC	VIN 2–3
	**N** = **40**	**N** = **12**
	**N (%)**	**N (%)**
**AGE (years)**		
<60	9 (22.5)	7 (58.3)
≥60	31 (77.5)	5 (41.7)
**DISTANT METASTASIS (lymph node**+**)**		
Yes	7 (17.5)	0
No	33 (82.5)	12 (100)
**LESION LOCATION**		
Labia Minora	10 (25.0)	3 (25.1)
Labia Majora	17 (42.5)	2 (17.7)
In both labia (Minora and Majora)	12 (30.0)	5 (41.7)
Clitoris and pubis	1 (2.5)	2 (16.7)

### Second harmonic generation (SHG)

Slides stained with H&E were submitted to the identification and selection of collagen (COL) in the stromal areas adjacent to VIN, in intratumoral stroma, and in normal adjacent tissues. In each case, three areas were selected, and these areas were submitted to SHG analyses at the INFABiC – Unicamp^14^.

SHG is a nonlinear optical process, in which two photons with the same frequency interact with a nonlinear material and generate a new photon with twice the energy and half the wavelength of the initial photons. Fibrillar collagen has high nonlinear susceptibility, with SHG being the ideal method to evaluate its structure^12,13^. SHG microscopy was performed on a confocal microscope Zeiss LSM 780-NLO (Carl Zeiss AG, Göttingen, Germany) using a lens with an objective of 40×/1.3 EC Plan-Neofluar with immersion oil. The highest numerical aperture (1.3) was necessary to allow an adequate spatial resolution to observe the fibrils. The excitation wavelength was 780 nm, with a pulse of approximately 100 fs at a repetition frequency of 80 MHz, supplied by a Tsunami Ti:Sapphire laser (Spectra-Physics, Irvine, CA, USA). The objective lens laser power was approximately 80 mW, with circular polarization. The acquisition time for each image was approximately 60 s.

Only the SHG forward signal at 390 nm was collected using a condenser lens 0.55 NA – WD 26 mm. A short-pass SP690 (Omega Filters, Brattleboro, VT, USA) was used to filter the excitation wavelength at 780 nm^25^; this was followed by filtering with long-pass filters LP490 at 45° to avoid two-photon excited fluorescence (TPEF), and SP430 at 90° to filter only the SHG signal. Two detectors were used to capture the TPEF and SHG signals, namely a photomultiplier tube detector (PMT) and non-descanned detector (NDD), respectively. Of these, only the SHG signal was considered for the present study. The emission spectrum analysis between 350 and 430 nm showed only a narrow peak at 390 nm consistent with the SHG signal. The TPEF signal given by eosin could also be detected at 530 nm.

The procedure described by Burke *et al*.^25^ was used, with daily optical alignments performed by obtaining a normalization factor, which consisted of the SHG image of a standard specimen (i.e., a histological section of human aorta), at the beginning of each experiment. The vision field of this objective was 212 × 212 cm, but to avoid the figure margins in which the SHG intensity was weaker, we used a zoom to select a more homogeneous area of 177 × 177 cm. To eliminate background signals, a “blind” observer, with a degree in physics and without medical training, applied a common threshold to all images, comparing them with the control sample of human aorta. After this, all background signals unrelated to COL were eliminated.

SHG signals were stratified according to COL organization and structure in the stroma, using image-pattern analysis methods. Quantitative analysis of COL parameters was performed on SHG images using ImageJ (http://imagej.nih.gov/ij/) and the OrientationJ plug-in software^26^. Nine representative areas (150 × 150 pixels) were randomly selected from each image to evaluate COL quantity, uniformity, and organization, as described below.

COL quantity was determined as the sum of SHG signal intensities in 1024 × 1024 pixel images, which was directly related to the quantity of collagen molecules. These values were obtained using the integrated density function of ImageJ software. Using the OrientationJ plug-in, as previously described^26^, we calculated COL uniformity (energy) and organization (coherence). Uniformity (energy) reflects the density of COL, i.e., whether fibers are farther from (lower values) or closer to (higher values) each other. Organization (coherence) reflects the spatial direction of COL, with values between 0 (completely isotropic areas, with zero directionality) to 1 (highly oriented fibers). From the perspective of mathematical definitions, these parameters should be correlated. The relationship between COL parameters for vulvar lesions, normal adjacent tissues, and VIN was added to the calculation^27^.

### Statistical analyses

Descriptive statistics for categorical variables are presented in tables as the distribution of absolute and relative frequencies, whereas continuous variables are presented as measures of central tendency (i.e., median). Normality was tested using the D’Agostino-Pearson test. The variables quantity and uniformity of collagen fibers were log-transformed, because they depend upon the square of the SHG signals. Continues values were plotted as the median and interquartile range (25th–75th). The Mann-Whitney nonparametric test was used to analyze numerical variables between two groups. The Kruskal-Wallis test was used to analyze numerical variables between three groups. Data analysis was performed using GraphPad Prism v 8.0 software (GraphPad Software, San Diego, CA). All comparisons were considered statistically significant at p < 0.05.

## Data Availability

Data sharing, evidence of data sharing and peer review of data required.
